# Microalgae Brewery Wastewater Treatment: Potentials, Benefits and the Challenges

**DOI:** 10.3390/ijerph16111910

**Published:** 2019-05-30

**Authors:** David Kwame Amenorfenyo, Xianghu Huang, Yulei Zhang, Qitao Zeng, Ning Zhang, Jiajia Ren, Qiang Huang

**Affiliations:** 1 Department of Aquaculture, Fishery College, Guangdong Ocean University, Zhanjiang 524088, China; davidamenorfenyo@yahoo.com (D.K.A.); yuleizhang88@163.com (Y.Z.); zeng364@163.com (Q.Z.); zning496@163.com (N.Z.); rjj12138@163.com (J.R.); 2 Guangdong Engineering Technology Research Center for Algae Breeding and Application, Zhanjiang 524088, China; 3 SDIC Guangdong Bio-Energy Co., Ltd., Zhanjiang 524025, China; huangqiang@sdiczn.com

**Keywords:** brewery industry, wastewater, microalgae, environmental protection

## Abstract

Concerns about environmental safety have led to strict regulations on the discharge of final brewery effluents into water bodies. Brewery wastewater contains huge amounts of organic compounds that can cause environmental pollution. The microalgae wastewater treatment method is an emerging environmentally friendly biotechnological process. Microalgae grow well in nutrient-rich wastewater by absorbing organic nutrients and converting them into useful biomass. The harvested biomass can be used as animal feed, biofertilizer, and an alternative energy source for biodiesel production. This review discusses conventional and current brewery wastewater treatment methods, and the application and potential of microalgae in brewery wastewater treatment. The study also discusses the benefits as well as challenges associated with microalgae brewery and other industrial wastewater treatments.

## 1. Introduction

Brewery industries, despite being a vital part of the producing country’s economy, consume large volumes of water during the production processes, and later release about 70% of it as wastewater [1,2]. Wastewater byproducts such as yeast surplus spent grains, produced from two main beer production stages (brewing and packaging) are the main contributors to environmental pollution when mixed with effluent [3]. Furthermore, flushing of human excreta, cleaning of floors, bottles, tanks and machines also contribute to the contamination of water bodies [3]. This effluent contains chemical oxygen demand (COD), nitrogen, phosphorous and other high organic loads that makes it unsuitable for any beneficial use [4]. Brewery wastewater may be discharged either directly into: (1) municipal sewers, (2) water bodies, or (3) the brewery’s wastewater treatment plant and (4) water bodies/municipal sewer system after pretreatment [5]. 

Discharge of untreated/partially treated brewery wastewater into water bodies raises environmental concerns. The major environmental concerns raised by the operation of breweries include water consumption, wastewater, solid waste and by-product generation, energy use and emissions to air. This phenomenon leads to environmental problems such as water scarcity, excessive growth of undesirable microbes that cause loss of aquatic lifeforms [6,7] and health-related problems in communities around the discharge areas [8]. There is therefore a need for brewery industries to adequately treat and manage their wastewaters before their final discharge into the environment. Conventional treatment methods; though extensively used in brewery wastewater treatment, usually generate huge amounts of sludge. Conventional treatment methods are also characterised by high operation and maintenance costs, which further makes them economically unfeasible. Additionally, the excessive use of chemicals may cause ecological imbalances. 

The use of microalgae, an environmentally friendly and cost effective water treatment method, has been identified as a way to address these problems [9]. Interestingly, microalgae have the potential to efficiently remove organic loads from wastewater, and provide a useful biomass byproduct. Currently, algae wastewater treatment and its biomass use are attracting attention worldwide [10]. Hence, this review discusses the characteristics of brewery wastewater, current treatment methods, and the application and potential of microalgae in brewery wastewater treatment. The study also discussed the benefits as well as challenges associated with the use of microalgae treatment methods to effectively treat brewery wastewater for the protection of the environment. 

## 2. General Characteristics 

Brewery wastewater is usually voluminous with high moisture content. This wastewater usually has high chemical oxygen demand (COD) and BOD due to the presence of organic components (sugars, soluble starch, ethanol, volatile fatty acids). The temperature of brewery wastewater usually ranges from 25 °C to 38 °C. Its pH levels are variable and dependent on the amount and type of chemicals used in cleaning and sanitizing (e.g., caustic soda, phosphoric acid, nitric acid, etc.). Nitrogen (N) and phosphorus (P) levels present are also dependent on the handling of raw material and the amount of yeast present in the effluent. Table 1 lists the main characteristics of brewery wastewater [11].

## 3. Current Wastewater Treatment Approaches 

### 3.1. Pretreatment Method of Brewery Wastewater 

Brewery wastewater is characterized by dark brown color, TSS, TS, etc. that requires pretreatment to minimize suspended particles and other organic loads. Generally, the brewery wastewater pretreatment process is meant to change the physical, chemical and or the biological properties of the feed water. The pretreatment process is carried out by physical, chemical or biological means or combinations of two or more methods. However, the selection of pretreatment method largely depends on the final discharge point of the effluent. For example, in a situation where the brewery does not discharge into the municipal drain, only primary and secondary treatments are required, but if the brewery is allowed to discharge into the municipal drain, pretreatment is required to reduce the organic loads of the municipal treatment plant and to also meet municipal wastewater treatment bylaws [12]. However, municipalities at times impose higher sewer discharge fees on the effluent volumes as well as organic loads and this may force some brewery industries to operate their own treatment plants in order to save cost.

### 3.2. Physical Treatment

Physical wastewater treatment has been used generally to reduce suspended solids from wastewater through sedimentation by gravitational force. The process also separates materials such as grease and oil from the effluent [13]. However, physical treatment methods only remove solid coarse materials but do not degrade pollutants. According to Thakur et al. [14] screening, flow equalization mixing, flotation and sedimentation are the physical methods currently used in effluent treatment. Flow equalization is a technique used to consolidate wastewater effluent in holding tanks for equalization before the wastewater is introduced into downstream treatment processes. Usually, physical treatment serves as a pretreatment stage of brewery wastewater treatment.

### 3.3. Chemical Treatment

Chemical treatment processes involve pH adjustment or coagulation/flocculation by adding different chemicals to the effluent to alter its chemistry [11]. Coagulation-flocculation is the first treatment step in the chemical wastewater treatment method. Flocculation involves stirring/agitation of chemically-treated effluent to induce coagulation that improves sedimentation performance by increasing particle size, thereby increasing settling efficiency [15]. Inorganic coagulants such as aluminum sulfate and ferric chloride have been widely applied in wastewater treatment [16]. During this treatment process wastewater, organic compounds are oxidized via the addition of chemical compounds like chlorine, ozone-oxygen, or permanganate to generate CO_2_, H_2_O and other inoffensive materials [17]. Chemical flocculants are highly efficient but are dangerous to human health and the environment [17]. The wastewater pH needs to be maintained between 6 and 9 in order to protect the microorganisms (bacteria) present. Usually, neutralization of wastewater pH using H_2_SO_4_ and HCl is not recommended due to their corrosive nature and the discharge limitation of sulfate and chloride [11]. However, the waste CO_2_ could be utilized as an acidifying agent to decrease alkalinity (high pH) of wastewaters before the anaerobic digestion. The *Detarium microcarpum* is reported to be an effective bio-coagulant for removal of turbidity from brewery effluent [16]. Okolo et al. [18], conducted a study on optimizing bio-coagulants for brewery wastewater treatment using response surface methodology. The method was used to evaluate the effects and the interactioin of three factors i.e., coagulants dosage, pH and the stirring time for solid particle removal on the treatment efficiency using *Detarium microcarpum* seed powder (DMSP) and oyster dried shell powder (ODSP) as coagulants. The results demonstrated the optimum conditions for coagulant dosage (100.53 mg/L), effluent pH (2.001) and stirring time (24.47 min) with 90.44% solid particle (SP) removal for DMSP and coagulant dosage (104.19 mg/L), pH (3.34) and stirring time (27.54) with 96.55% SP removal for ODSP.

### 3.4. Biological Treatment Approaches

Brewery effluent requires efficient treatment methods that can break down the organic loads in the wastewater. The effluent is passed through both anaerobic (using aerobic bacteria) and aerobic (using activated sludge) digestion processes aiming to reduce effluent’s COD before its discharge into the municipal sewer.

#### 3.4.1. Anaerobic Process

Anaerobic digestion is a natural process in which various microbial species work together in the absence of oxygen to transform organic wastes through a variety of intermediates into biogas [19]. This treatment method is widely used in brewery because the generated biogas may be used to maintain operational temperature or to generate revenue. Anaerobic digestion, however, is influenced by a number of factors, including nutrients, organic loads, carbon/nitrogen ratio, temperature and pH of the wastewater [20,21].

An optimum temperature is required by anaerobic bacteria to effectively digest organic pollutants [22,23]. Usually, most commercial anaerobic plants operate in the mesophilic range [24]. However, the working bacteria are classified based on their optimum pH range. Acidogenic and methanogenic bacteria work perfectly at optimum pH values of less than 6.0 and 7–8, respectively. [23,25]. This treatment method has notable advantages and disadvantages which are listed in Table 2.

#### 3.4.2. Aerobic Treatment

The aerobic treatment method takes place in the presence of oxygen by aerobic microorganisms (bacteria) that metabolize organic matter in the wastewater. They produce more microorganisms and inorganic end-products such as carbon dioxide, ammonia and water. Aerobic processes are more efficient in the digestion of pollutants [27]. In the aerobic biological treatment process, microorganisms convert non-settleable to settleable solids, followed by sedimentation which allows the settleable solids to settle and separate out. The widely used aerobic treatment method in wastewater treatment include: (1) activated sludge process, (2) attached growth (biofilm) process (3) trickling filter process. 

However, the choice of aerobic treatment methods strongly depends on the strength of pollutants in the effluent. Usually activated sludge and trickling filter processes are used in brewery wastewater treatment due to the strong organic pollutants in the effluent. 

Activated sludge processes are mostly used in wastewater treatment. In this process, the wastewater flows into an aerated agitated tank that is primed with activated sludge. The suspension of aerobic bacteria in the aeration tank is mixed vigorously by aeration devices, which supply oxygen to the biological suspension [11]. 

In the attached growth process, the aerobic biological process creates a favorable environment for microorganisms that desire to attach to the solid surface [27]. During the trickling filter process, effluent from the brewery is sprayed on the surface of solid materials like gravel, stone/plastics, and these materials allow the effluent to trickle down via decomposed microorganism media. Application of both anaerobic and aerobic processes in wastewater treatment is associated with huge capital cost. However the operational cost of the anaerobic process is comparatively lower than that of the aerobic treatment. Aerobic processes are also hindered by the physical and chemical variation of the wastewater [28], high cost of treatment and the formation of excessive sludge by the microorganisms [29]. Table 3 shows a comparison between aerobic and anaerobic treatments.

### 3.5. Membrane Filtration

The membrane filtration process uses semi-permeable materials that allow certain molecules to pass through them. Membrane filtration techniques such as reverse osmosis (RO), nanofiltration (NF), ultrafiltration (UF) and microfiltration (MF) have been applied significantly in brewery effluent remediation and can result in 99% removal of COD, BOD, and TSS [2,31]. Efficient membranes are characterized by high pollutant rejection rates, great durability, high permeate flux, low maintenance cost and high resistance to chemicals [32]. Membrane filtration is considered safe and environmentally friendly [33]. Membrane filtration has been known for its efficient removal of physical, microbial and chemical pollutants compared with other systems, hence it forms an integral part of the drinking water treatment process [34].

NF was used by Braeken et al. [2] to treat brewery wastewater for recycling; the results of the study showed that NF efficiently removed COD, Na^+^, and Cl^−^ with an average removal rate of 100%, 55%, and 70%, respectively. Also, several studies on the application of RO reviewed by Madaeni and Mansourpanah [35], revealed that RO may decrease the COD of the effluent by more than 90% or even completely. Madaeni and Mansourpanah [35] biologically treated alcohol wastewater (which is similar to that of brewery wastewater since both productions involved fermentation) from a manufacturing plant by various polymeric RO and NF membrane with COD range of 900 to 1200 mg·L^−1nv^. A polyethylene terephthalate RO membrane yielded magnificent results with higher flux (33 kg m^−1^ h^−1^) while COD was completely removed (100%). In another test conducted on brewery bio-effluent, using an internal aerobic membrane bioreactor (internal MEMBIOR), the effluent’s COD varied strongly (from 1500 to 3500 mg·L^−1^) during the treatment process but was later reduced to about 30 mg·L^−1^ at the end of the treatment. The membrane also retained the suspended solid completely making the effluent suitable for reuse [36]. The main challenges for this technology are fouling and high energy consumption. More research should be focused on anti-fouling and less energy consumption membrane filtration methods for efficient treatment of brewery effluent. 

### 3.6. Membrane Bioreactor Treatment 

A membrane bioreactor treatment (MBR) is the combination of two treatment technologies that are membrane filtration and advanced biological treatment (activated sludge or an anaerobic unit). This technology has produced positive results in wastewater treatment over the past decade [37,38].

Increasing water prices and scarcity has called for combination of treatment technologies that can effectively treat wastewater for its reuse. MBR is noted as an economical and technically feasible choice of wastewater treatment [39]. Descriptively, MBR is a system that integrates membrane with a bioreactor. Submerged and side-stream configurations are the two main recognized MBR systems. In a submerged process, the membrane is placed inside the reactor and then submerged into mixed liquor. In a sidestream process, the membrane unit is positioned outside the reactor and the reactor mixed liquor flows over a recirculation loop containing the membrane. However, sidestream MBRs are more energy intensive than submerged MBRs due to their high operational transmembrane pressures and the substantial volumetric flow needed to attain the preferred cross flow velocity [40].

The technology was applied by Dai et al. [41] in brewery wastewater treatment using an upflow anaerobic sludge blanket (UASB) reactor with an integrated membrane. The result showed successful removal of COD by 96%. There are extensive published articles on the application of MBR technology in brewery wastewater treatment with almost all reporting significant levels of COD removal rate by 90% or more [42,43,44]. This has demonstrates that the MBR process can be an effective method for brewery wastewater treatment. Nevertheless, MBR technology is challenged by factors such as (1) fouling that needs to be addressed by regular cleaning and maintenance, (2) high capital cost due to the combination of more than one treatment methods (membrane and aerobic/anaerobic reactors), (3) high energy consumption leading to extra costs.

### 3.7. Advanced Oxidation Treatment Process

Advanced oxidation treatment processes (AOPs) are widely used in the treatment of both distillery and brewery wastewater. In this process, hydroxyl radicals (•OH) are produced by the use of ozone, hydrogen peroxide and ultraviolet irradiation in the first stage of the oxidation. In the second stage, organic loads react with hydroxyl radicals to produce precipitates. AOP technologies can be made possible through the combination of the hydrogen peroxide/ultraviolet irradiation (H_2_O_2_/UV), zone/ultraviolet irradiation (O_2_/UV) and ozone/hydrogen peroxide (O_2_/H_2_O_2_) [45]. Ozone and hydroxyl radicals (•OH) are robust oxidants and can oxidize many organic compounds. Ozone reacts with an appreciable number of organic compounds when dissolved in water, thereby aiding in the removal of removal of organic contaminants from wastewater. It reacts directly or indirectly in the oxidation process, directly as molecular ozone and indirectly by the production of secondary oxidants in the form of free radical species such as hydroxyl radicals (•OH) [45]. Fenton’s oxidation is another known AOP process based on the Fenton reaction. This process is a combination of hydrogen peroxide/ion salts (Fe^2+^ or Fe^3+^) [46]. Fenton oxidation technology produces hydroxyl radicals (•OH) that result in precipitate formation and decolorization of effluent. Fenton technology produces a homogeneous reaction that is ecologically friendly [46]. A further search on AOP processes showed a few other combination such as TiO_2_/U, boron-doped diamond electrodes and catalytic ozonation. However these processes are still on laboratory scale utilization stage. Application of AOP in brewery and other wastewater treatment showed positive results and have the potential for future brewery wastewater treatment. However the technology may require supplementary treatment to eliminate ozone and this may increase the treatment cost. Also, the AOP processes are challenged by turbidity and NO_3_ which needs to be addressed.

### 3.8. Air Cathode Microbial Fuel Cells Treatment

Recently, microbial fuel cell (MFC) wastewater treatment method has drawn worldwide attention due to its potential to convert organic pollutants into electricity whilst simultaneously purifying effluent. MFC reactors are combined with anaerobic treatment characteristics; that is using microorganisms to digest organic pollutants close to the anode, with the cathode exposed to oxygen. Electrons (released by bacterial oxidation of the organic loads) are transferred via the external circuit to the cathode, where they combine with oxygen to form water [47]. For example, Feng et al. [47], evaluated the efficiency and suitability of the MFC process in brewery wastewater treatment. According to the authors, for an effective MFCs process, there is the need for a good understanding of the how operational factors and the solution chemistry influence treatment efficiency. Furthermore, the authors evaluated the efficiency of MFC by examining maximum densities, and removal of COD as functions of effluent strength, temperature and columbic efficiencies (CEs). The result showed a reduction of maximum power density from 205 mW/m^2^ to 170 mW/m^2^ when the temperature was reduced from 30 °C to 20 °C. Nevertheless, there was slight decrease in COD removal and CEs with decreasing temperature. Moreover, the buffering capacity strongly affected the efficiency of the rector. COD removal rate was 85% at 20 °C and 87% at 30 °C. This technology can be used as a new method of brewery effluent treatment.

### 3.9. Activated Carbon-Based Treatment 

Activated carbon is widely used in municipal drinking water purification, point-of-use (POU) and point-of-entry (POE) filters and industrial wastewater purification. Activated carbon possesses strong adsorbents which aid in the removal of a variety of organic compounds from industrial wastewater. Carbon may be used to purify the total flow of an effluent containing different pollutants or can be used as part of a multistage approach; that is to remove specific pollutants present in the effluent [11]. Brewing processes such as fermentation contribute to an unpleasant odor of the effluent. This effluent may contain some molecules with aromatic and carbon-sulfur bonds that usually produce bad smell and taste. These molecules especially adhere to carbon. Moreover, carbon may also serve as a dechlorination agent due to its ability to react with oxidizing agents like hypochlorous acid and chlorine dioxide [11]. Carbon adsorbent is used to treat tannic acid to remove odor from brewing. Activated carbons can be an odorless wastewater treatment option in the brewing industry. This technology is less expensive and does not need electricity/ high water pressure. However, activated carbon is unsuccessful against many inorganic pollutants such as iron, salts, fluoride, aluminum and calcium [11]. Table 4 shows COD removal of various treatment methods. 

### 3.10. Microalgae Treatment Method

**Figure 1:**
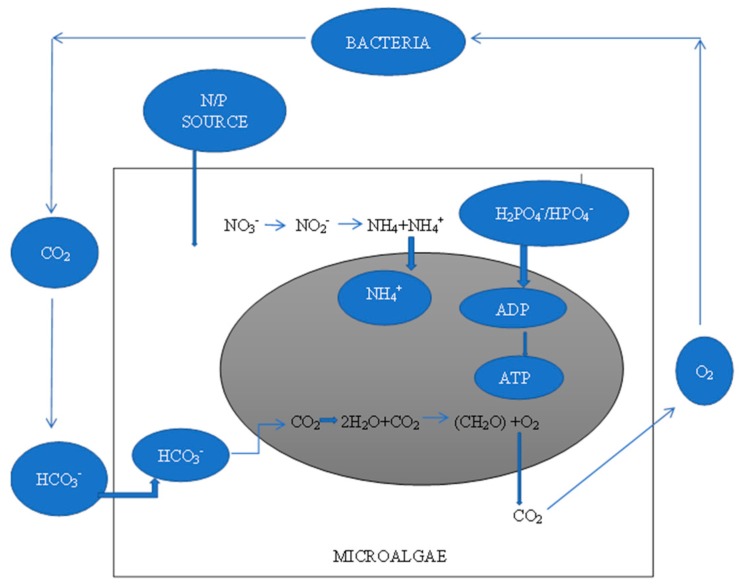
A bacterial-microalgae relationship in wastewater treatment.

Microalgae are considered to be one of the favorable wastewater agents due to their ability to absorb nutrients and convert them to biomass [50]. During the brewery wastewater treatment, nitrogen, phosphorus and other nutrients present in the wastewater are adequately absorbed by microalgae for their growth. Microalgae, through their photosynthetic activities, freely release oxygen which is utilized by bacteria in the wastewater. Microalgae also fix CO_2_ by assimilating HCO_3_ from CO_2_ via respiration. Figure 1 shows the mechanism of the bacteria-microalgae relationship in wastewater.

**Figure 2:**
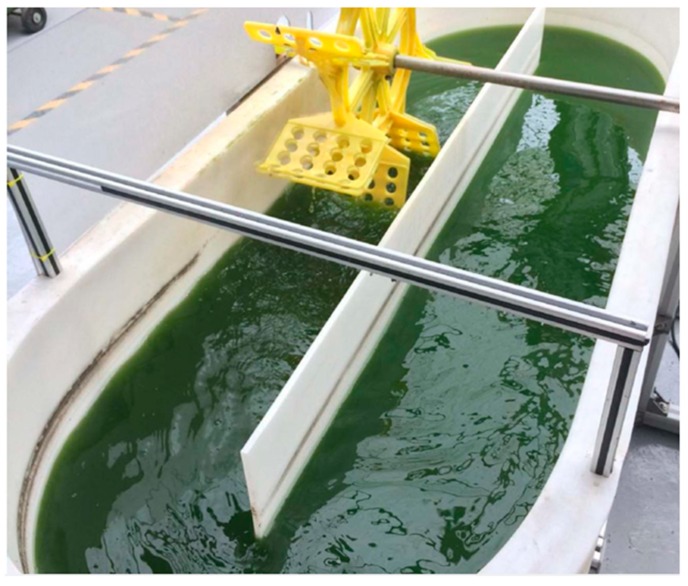
Example of a raceway pond wastewater treatment [59].

**Figure 3:**
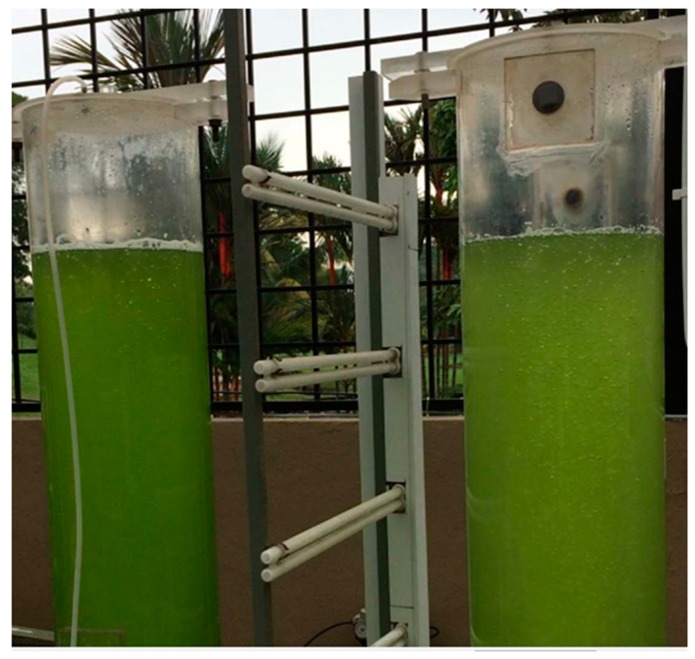
Model of tubular photobioreactors [59].

Until recently, the application of microalgae in wastewater treatment had only been restricted to the laboratory. Raceway ponds and photobioreactor technologies have been applied in microalgae wastewater treatment, including brewery wastewater. Raceway ponds are semi-circular at the two ends, with a shallow open system. The system has paddle wheels that provide continuous mixing of the microalgae in the wastewater for nutrients and sunlight [51]. A raceway pond is depicted in Figure 2. Photobioreactors are constructed either in vertical and horizontal columns. The structure allows penetration of light to the microalgae. CO_2_ is sparged in and circulated to allow microalgae to have access to enough CO_2_ [52]. Figure 3 shows the model of the tubular photobioreactor.

A study by Lutzuet et al [53] demonstrated that microalgae (*Scenedesmus dimorphus)* was able to remove more than 99% of both nitrogen (N), and phosphorous (P) from brewery wastewater within one week; nitrogen was reduced from the initial concentration of 229 mg·L^−1^ to a final concentration below 0.2 mg·L^−1^ and phosphorous initial range of 1.4–5.5 mg·L^−1^ to the final concentration lower than 0.2 mg·L^−1^.

Similarly, Ferreira et al. [54] concluded in their report that *Scenedesmus obliquus* removed almost all the pollutants present in the various wastewater (poultry, swine and cattle breeding, brewery and dairy industries, and urban). Subramaniyam et al. [55] cultivated *Chlorella* sp. in brewery wastewater and concluded that *Chlorella* sp. removed total nitrogen, phosphorus, and organic carbon completely with substantial growth of the microalgae (*Chlorella* sp.). 

Luo et al. [56] also determined the nutrient removal efficiency by *Desmodesmus* sp. CHX1 in piggery wastewater and reported that *Desmodesmus* sp. CHX1 removed 78.46% of nitrogen and 91.66% of phosphorus. Another study conducted by Duan et al. [57], compared the biochemical compositions of four microalgae (*Nannochloropsis oceanica*, *Auxenochlorella pyrenoidosa*, *Arthrospira platensis*, and *Schizochytrium limacinum*) and four macroalgae (*Ulva prolifera*, *Saccharina japonica* (*Areschoug*), *Zostera marina*, and *Gracilaria eucheumoides* Harvey) and arrived at the conclusion that, the nitrogen and phosphorus contents in the algal biomass ranged from 1.24 to 10.79% and 0.03% to 2.49%, respectively, and confirmed the nutrient absorption capability of microalgae from wastewater. Travieso et al. [58], conducted a study on the efficacy of distillery effluent treatment by the microalga *Chlorella vulgaris.* The authors concluded that *Chlorella vulgaris* reduced more than 98% of COD and BOD, and the final effluent was safe to be discharged into the environment.

Though the microalgae method is capable of removing high amount of pollutants from brewery effluent, the technology is limited in terms of salt, odor and color removal. This technology requires a combination with other cost effective method(s) for total removal of contaminants from the effluent. Microalgae-based wastewater treatment technology can be combined with membrane technology for the polishing treatment stage. Light and temperature are one of the limiting factors of algae-based wastewater treatments. Microalgae require optimum light and temperature for growth. This technology may therefore not be applicable in temperate regions due to the relatively low sunlight and temperature. Alternatively, artificial lighting systems could be used in these countries which may increase the cost of treatment. However, the biomass can be processed into biofuel and other useful products.

## 4. Potential of Microalgae in Wastewater Treatment

### 4.1. Cost-Effectiveness

The cost of maintaining microalgae growth in wastewater is lower than that of conventional wastewater treatments. Organic loads found the brewery wastewater are suitable for the growth of microalgae, thereby making it an extremely attractive means for sustainable and low-cost wastewater treatment [60,61,62]. Several species of microalgae are able to capture nutrients from wastewater. The capital cost of this process is less expensive as compared to conventional wastewater treatment processes [63]. 

### 4.2. Low Energy Requirement

Microalgae release oxygen as a byproduct during wastewater treatment and this is utilized by aerobic bacteria to further degrade the remaining organic loads. This reduces the energy cost compared to the cost of mechanical energy for aeration during conventional wastewater treatment. Approximately, 1 kWh of electrical power is needed to remove 1 kg of BOD in the activated sludge process. During this process, 1 kg of fossil carbon dioxide is produced from power generation. Microalgae do not require any energy input to remove 1 kg of BOD from brewery wastewater and produce 1 kWh of electric power through methane production by algal biomass [64].

### 4.3. Reductions in Sludge Formation

The primary objective of every wastewater treatment plant is to reduce or eliminate sludge. Conventional wastewater treatment is characterized by the use of large amounts of chemicals. Substantial use of chemicals may result in the formation of sludge. This produces hazardous solid wastes which must be disposed of into the environment. Microalgae wastewater treatment requires no chemical additives and sludge is accumulated in a form of algal biomass [63].

### 4.4. Greenhouse Gas Emissions

Global warming is of great concern worldwide. According to Wang et al. [65] CO_2_ mitigation has been strategically tackled in two ways; chemically and biologically. Chemical approaches involve separation, transporting and sequestration. These approaches are energy consuming and costly, therefore there is a need for alternative cost-effective and sustainable means to curb the threat.

Microalgae are one of the emerging biotechnological approaches to mitigate CO_2_ and about 2,000,000 species are useful for CO_2_ sequestration [66]. Fixation of CO_2_ through photoautotrophic algal culture has the capacity to decrease CO_2_ in the atmosphere. Approximately, microalgae fix 183 tons of carbon dioxide to produce 100 tons of biomass [67]. Microalgae grow more rapidly than other terrestrial plants due to their ability to capture solar energy more efficiently [59]. Li et al. [68], revealed that microalgae have much higher growth rates and CO_2_ fixation abilities compared to conventional forestry, agricultural, and aquatic plants.

In addition, microalgae require carbon dioxide for growth and, any source of CO_2_ can be used for cultivating algae. However using pure CO_2_ may be very expensive and using air does not require transport, but the amount of CO_2_ (~0.04 w%) contained in the air may not be adequate for the growth of microalgae. A sufficient amount of CO_2_ must be supplied to enable optimal algae growth [69,70]. CO_2_ (flue gas) from industrial production can be utilized to address these challenge [69]. Usually, flue gas contains a huge amount of CO_2_, but the actual concentration is dependent on the process and the origin. For example, flue gas from coal-fired power plants are lower in CO_2_ concentration compared with flue gas from natural gas-fired power [71]. However, one must be prepared to address the difficulties associated with utilization of CO_2_ from industries like transportation of flue gas to the treatment site, by siting race way ponds or photobiorectors close to the flue gas production industries.

**Figure 4:**
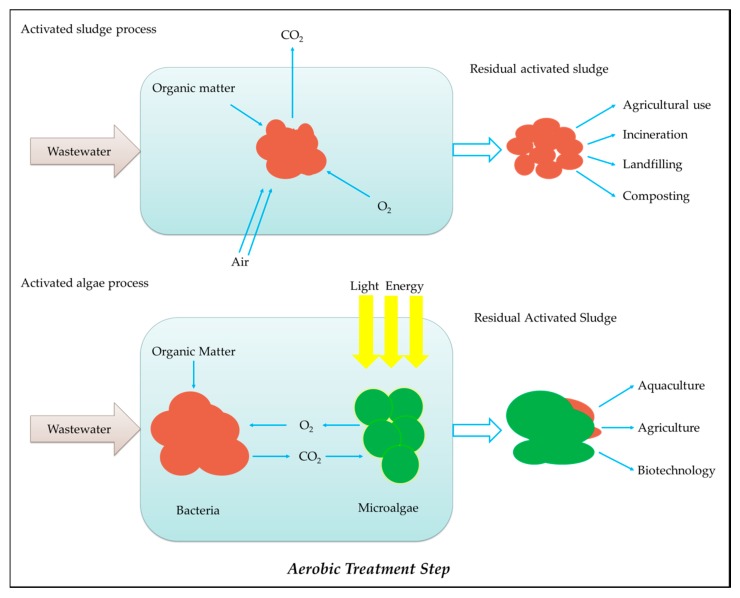
CO_2_ savings comparison between activated microalgae and conventional activated sludge processes in aerobic wastewater treatment.

In comparison, as shown in Figure 4, microalgae-based wastewater treatment reduces tons of CO_2_ compared to conventional treatment methods. For example, raceway ponds or high rate algal pond (HRAP) systems reduce 100 to 200 tons of CO_2_ per ML of treated wastewater by utilizing bacteria, sunlight and photosynthesis compared to electromechanical treatment in a conventional oxidation pond.

Furthermore, assimilation of nitrogen by algae could reduce an additional tons of CO_2_ (100–200) per ML [72]. Hence, microalgae-based wastewater treatment or when it is integrated into other wastewater treatment plants can biologically mitigate CO_2_ levels. This method is more economical, cost-effective and eco-friendly [73,74].

## 5. Benefits of Microalgae-Based Wastewater Treatment

### 5.1. Fertilizers and Bio-Fertilizers

Excessive application of inorganic fertilizers alters soil fertility by increasing and decreasing soil acidity and pH, respectively. Also, inorganic fertilizers contain substances such as nitrates and phosphates that are subsequently washed into water bodies by rains and sewage that may lead to eutrophication. Microalgae play a useful role in the agro-industry. The harvested algal biomass from the wastewater may be processed into plant fertilizers. These fertilizers improve the mineral composition and water holding capacity of agricultural soils [75]. According to Hasyim et al. [76], irrigation using raw brewery wastewater as fertilizer source leads to low plant growth thereby resulting in low yield and poor soil condition. Microalgae can also fixed nitrogen into the soil. *Nostoc* sp., *Scytonema* sp., *Aulosira* sp., *Toplythrix* sp, and *Plectonema* sp. fix nitrogen into the soil and are usually utilized as bio-fertilizers [77]. 

### 5.2. Animal Feed 

Microalgae serve as live feed in the aquaculture industry due to their nutritional contents and easy digestibility. Microalgae are composed of (dry matter) 39–71% of protein, 10–57% of carbohydrates, mainly polysaccharides, cellulose, and starches [78]. Harvested biomass may be used directly or indirectly to feed oyster, shrimp, and bivalve larvae. Microalgae can also enrich zooplankton for feeding fish. Phang et al. [79] revealed that the biomass composition of *Spirulina* cultured in a HRAP for the treatment of sago wastewater may be used as high-quality animal feed. Microalgae biomass is viable as a partial replacement in poultry feed for conventional proteins and carotenoids to enhance the yellow color of broiler skin and egg yolk [80]. Several studies have suggested incorporation of treated wastewater-based microalgae biomass into animal feed. However, it has received little attention due to public perception and quality food regulations on animal feeds.

### 5.3. Bio-Fuel Production

A biofuel can be defined as a substance (biohydrogen, biodiesel, bioethanol, biomethanol) with a large heat of combustion value obtained from biomass [81,82]. There is a growing demand for energy globally. Approximately, 80% of consumed energy is obtained from fossil sources [83]. However, fossil fuel extraction and use increases greenhouse gas emissions in the atmosphere [84] which leads to global warming. In recent times, studies have been focused on alternative energy sources due to the drawbacks associated with fossil fuel. Biofuels have gained significant consideration and have been regarded as a promising source of alternative energy source.

Microalgae are said to be the most promising source for biofuel production. This is because they have a high growth rate and high photosynthetic efficiencies [85]. First generation biofuels raised arable land shortage-related concerns and this may in turn cause a shortage of food globally. Oil-plants such as palm, soybean and rapeseed are of interest in recent times but a large arable land area is used for their cultivation [86]. Second generation biofuels use *Jatropha curcas*, but its slow growth rate coupled with the high use of arable lands makes it unprofitable. However, microalgae biofuel is claimed to yield 10–100 time more fuel per unit area than other biofuel sources (conventional crops) such as soya bean, and oil palm [87]. In addition, microalgae do not have any arable land-related issues [66] as microalgae can be grown in the brewery and other industrial wastewater.

## 6. Challenges Associated with Microalgae Wastewater Treatment

### 6.1. Pre-Treatment of Wastewater

Raw brewery wastewater contains high levels of contaminants such as bacteria, protozoa, fungi and solid particles that inhibit the growth of microalgae. These organisms compete with microalgae in the wastewater for nutrients and other minerals. The wastewater requires pretreatment to eliminate all organisms before the introduction of microalgae. In recent times various pretreatment technologies have been applied to regulate large volume of wastewater (brewery wastewater). Pretreatment methods such as filtration and autoclaving are widely used. However, studies revealed that, autoclaving has been noted to be the most effective pretreatment method for microbial elimination [88], but the authors also stated in the same report that autoclaving may interfere with the nutrient content of the wastewater [88]. This hypothesis was tested and proven by Cho et al. [89]. The authors observed high biomass concentrations for the filtration method compared to the autoclaving method when microalgae were cultured in municipal effluent. This shows that the particulates present in the autoclaved pretreated effluent may have prevented the microalgae from having full access to light utilization for photosynthetic activities. Application of these pretreatment methods on a commercial basis may not be feasible due to the high energy cost involved. However, other alternative wastewater pretreatment methods have been reported. For example, ultraviolet (U.V) and chlorination have been noted for wastewater pretreatment and were effectively utilized by Qin et al. [90]. 

### 6.2. Selection of Suitable Microalgae Strain for Brewery Wastewater Treatment

Selection of microalgae species for the brewery and other wastewater treatment is very vital. Due to the physical and chemical composition of brewery wastewater, microalgae species should be robust enough to deal with fluctuations in environmental factors. Also, the species should have the ability to share metabolites to accommodate stress, override any attack of unwanted species and nutrient limitations [91]. In brewery wastewater treatment only a few microalgae species (Table 5) have been reported to be wastewater tolerant.

### 6.3. Harvesting of Microalgae from Wastewater

One of the problems connected with microalgae brewery and other industrial wastewater treatment is the separation of microalgae from the effluent. This process is said to be energy intensive. The harvesting techniques include flocculation, centrifugation, flotation, gravity sedimentation, filtration and ultrasonication. Factors such as rapid growth rate, small portion of algae in the total suspension, microscopic size of a single cell and negative cell surface charge that prevents them from forming larger and easily harvestable particles complicate harvesting of microalgae [93]. These combinations affect normal separation methods like sedimentation, filtration and microstraining [94] hence increases the cost of harvesting algal biomass. Table 6 shows various algae biomass harvest techniques with their advantages and disadvantages. It is however necessary for more research on finding more simple, cost-effective and efficient method of harvesting algae biomass from wastewater. 

### 6.4. Internal Shading

Internal shading limits the photosynthetic activity of microalgae. Brewery wastewater is rich in nutrients and microalgae can multiply rapidly within a day (24 h) in this effluent, but its multiplication within the log phase may be short (3.5 h) [66,97]. The speedy increase in cell number may decrease the amount of light access by portion of the effluent because the dense culture in the upper part limit the strength of light that penetrates into the water [98]. Raceway ponds or photobioreactors may be used to solve this problem. In raceway ponds, the bottom portion of the microalgae is circulated close to the surface for the microalgae to capture light energy by the rotation of the shift paddle in the culture media. In a photobioreactor system, light is set close to the upper portion of photobioreactor and air is sparged into the system to rotate the bottom portion of the microalgae close to the surface to allow the microalgae to capture light energy.

### 6.5. Suspended Solids and Turbidity of the Waste Water

Brewery and other industrial wastewater contain a significant amount of suspended solids. This may interfere with the growth process of the microalgae. High turbidity of the wastewater also limits penetration of light through the wastewater; which affects the photosynthesis process of the microalgae [99]. However, pre-treatment wastewater methods like flocculation can be employed in order to reduce suspended solids in the wastewater. Addition of turbulence to the effluent can also be used to address this problem by exposing the microalgae in the effluent to light in a short period thereby increasing the productivity of the microalgae-based wastewater treatment. 

## 7. Conclusions

As the brewery industry continues to expand, the amount of wastewater it produces will continue to increase and thus its negative impact on the environment. In order to safeguard the environment, biological methods such as aerobic and anaerobic treatments are mostly used due to their capability to remove high organic loads and COD. However, these methods are associated with high capital and operating costs. Moreover, these methods are only applied as pre-treatment options and the water may require further treatment. 

In this review, MBR, activated carbon and MFC methods have shown some promising results that have great potential for brewery wastewater treatment. However, high energy consumption and maintenance cost may be an inhibitory factor. Membrane filtration is being used for industrial brewery effluent and other industrial wastewater treatment. The technology is also being applied in drinking water and wastewater reuse. This technology has undergone speedy improvement in terms of quality and costs in recent times and could be used as a polishing step after microalgae treatment. Activated carbon-based treatment methods are less expensive, efficient in organic pollutant removal and can be a suitable treatment option for the brewery industry but, it may be faced with environmental and health concerns due to the use of carbon/coal for the treatment of effluent on a large scale. 

This study has shown some promising outcome from microalgae treatment methods. Microalgae treatment has high potential in brewery wastewater treatment. The technology is reliable, eco-friendly, and cost effective. Moreover, it is effective in removing ammonia and phosphorus from brewery effluents. Microalgae wastewater treatment has numerous benefits that have been outlined in this article. However, this technology requires the integration of other treatment methods in order to improve upon the final effluent for the protection of the environment. Currently, there are not many works about these integration options. This requires urgent investigation of other treatment methods with microalgae-based treatment methods, especially regarding color and odor removal from brewery effluent for its possible reuse. Further scientific research should be focused on finding more microalgae strains that are robust enough to adapt to stress and other growth inhibitors to effectively treat brewery and other industrial wastewaters for total protection of the environment.

## Figures and Tables

**Table 1:** Characteristics of the brewery wastewater [11].

Parameter	Value
pH	3–12
Temperature (°C)	18–40
BOD (mg·L^−1^)	1200–3600
COD (mg·L^−1^)	2000–6000
Phosphorus (mg·L^−1^)	10–15
TKN (mg·L^−1^)	25–80
TS (mg·L^−1^)	500–8750
TSS (mg·L^−1^)	2901–3000
TDS (mg·L^−1^)	2020–5940
VFA (mg·L^−1^)	1000–2500

BOD: Biochemical oxygen demand, TKN: Total Kjehldahl Nitrogen, TS: Total Solids, TSS: Total Suspended Solids, TDS: Total Dissolved Solids, VFA: Volatile Fatty Acids.

**Table 2:** Advantages and disadvantages of anaerobic waste treatment processes.

Advantages	Disadvantages
1. Low sludge production	1. Long start-up and retention times
2. Low nutrient (nitrogen and phosphorus) requirement	2. Requires high temperatures for effective operation
3. Low capital cost and operating costs	3. Requires monitoring for smooth operation
4. Production of methane, a source of energy	4. Shock and variable load can upset microbial balance
5. Production of liquid and solid residues that may be used as soil conditioners	5. Usually used as a pretreatment stage.
6. Inactivation of pathogens present in the waste	6. Aerobic ‘polishing’ may be required before discharge to the aquatic environment
7. Survival of microbial biomass in anaerobic treatment reactors for long periods of little or no feeding	

Adapted from Malina and Pohland [26].

**Table 3:** Comparison between aerobic and anaerobic systems.

	Aerobic	Anaerobic
COD removal rate (%)	90–98	65–90
Nitrogen/Phosphorus removal	High	Low
Energy production	Co_2_ is released (Low)	High production of biogas (CH_4_)
Energy Consumption	High	Low
Sludge production	High	High solid retention (Low)
Space requirement	High	Low
Discontinuous operation	Challenging	Low

Adapted from Driessen and Vereijken [30].

**Table 4:** Treatment methods used for brewery effluent and the efficient removal of chemical oxygen demands (CODs).

Process	COD Removal Rate (%)
Microbial fuel cells	94 [48]
Membrane bioreactor	96 [41]
Nanofiltration	96 [2]
Upflow anaerobic sludge blanket reactor	73–91 [49]
Reverse osmosis	100 [49]

Adapted from Simate et al. [11].

**Table 5:** Microalgae species used in brewery wastewater treatment by various authors.

Microalgae Species	References
*Scenedesus obliqus*	[54]
*Chlorella vulgaris*	[92]
*Chlorella prototheoides*	[92]
*Scenedesmus dimorphu*	[53]

**Table 6:** Harvesting techniques of microalgae biomass.

Technique	Advantages	Disadvantages
Flotation	-Able to process large volumes of biomass as air bubbles adhere tomicroalgae, making them buoyant	-Contamination with flocculation agent
Filtration	-Effective recovery for small sized microalgae	-High cost, algal species specific and clogging/fouling of filters
Centrifugation	-Rapid and efficient with 95% removal efficiency	High energy and maintenance cost
Gravity sedimentation	Low cost and energy efficient as microalgae biomass are left to settle naturally	Takes long time to settle and ineffective for small sized microalgae
Ultrasonication	-Can operate continuously	-Safety problem, disrupted cells unsuitable for further processing
Flocculation	-Cost effective	-Biomass unsuitable for further use (e.g., animal feed or anaerobic digestion); chemical flocculant contamination

Source: [95,96].
